# Attention Trade-Off for Localization and Saccadic Remapping

**DOI:** 10.3390/vision5020024

**Published:** 2021-05-20

**Authors:** Anna Dreneva, Ulyana Chernova, Maria Ermolova, William Joseph MacInnes

**Affiliations:** 1 Faculty of Psychology, Lomonosov Moscow State University, 125009 Moscow, Russia; 2 Vision Modelling Laboratory, Faculty of Social Science, HSE University, 101000 Moscow, Russia; umchernova@edu.hse.ru (U.C.); jmacinnes@hse.ru (W.J.M.); 3 School of Psychology, HSE University, 101000 Moscow, Russia; ermmmaria@gmail.com; 4 Department of Neurology & Stroke, Hertie Institute for Clinical Brain Research, University of Tübingen, 72074 Tübingen, Germany

**Keywords:** attention, dual task paradigm, remapping, retinotopic mapping

## Abstract

Predictive remapping may be the principal mechanism of maintaining visual stability, and attention is crucial for this process. We aimed to investigate the role of attention in predictive remapping in a dual task paradigm with two conditions, with and without saccadic remapping. The first task was to remember the clock hand position either after a saccade to the clock face (saccade condition requiring remapping) or after the clock being displaced to the fixation point (fixation condition with no saccade). The second task was to report the remembered location of a dot shown peripherally in the upper screen for 1 s. We predicted that performance in the two tasks would interfere in the saccade condition, but not in the fixation condition, because of the attentional demands needed for remapping with the saccade. For the clock estimation task, answers in the saccadic trials tended to underestimate the actual position by approximately 37 ms while responses in the fixation trials were closer to veridical. As predicted, the findings also revealed significant interaction between the two tasks showing decreased predicted accuracy in the clock task for increased error in the localization task, but only for the saccadic condition. Taken together, these results point at the key role of attention in predictive remapping.

## 1. Introduction

When performing our everyday activities, we can easily attend several objects at a time even though some of them are located outside the fovea. However, if one particular object or task captures critical attentional resources, processing other objects or tasks may be reduced. Our attention has a limited capacity [1], so it selects and prioritizes tasks according to the needs of the moment. We are able to attend to more than one task, but interference degrades performance in dual tasks when both tasks share the same resource [2]. The load theory of attentional selection [3,4,5] further suggested a direct trade-off such that as attended items require more perceptual resources, then unattended items or distractors get less resources and vice versa. This can be interpreted as a competition for resource allocation between different cognitive processes with executive control needed in cases of irrelevant distractors. For instance, perceptual load may be implicated in phenomena such as attentional blink (when an observer fails to detect the second target if it occurs within 200–500 ms after the first), inattentional blindness (when an observer fails to detect a stimulus because of the attentional lack rather than any vision deficits), or inattentional deafness (which refers to neglecting unexpected auditory information) under high visual perceptual load when the amount of information in processing the task is considerable [6,7].

Saccades also require attention and the target location of any saccade receives the benefit of attention [8] and attention may even precede the arrival of gaze [9]. Saccades and covert attention do share common resources and studies have shown a trade-off between covert spatial attention and saccade preparation [10,11]. Indeed, it has been proposed that eye movements and attention are simply different degrees of activation within the same mechanism [12,13] but this proposal has limitations, such as the observed independence between endogenous attention and oculomotor activation [14,15].

The retinal image changes with each eye movement, however, we are able to maintain a stable percept of the visual world. Early visual areas follow a retinotopic organization [16,17,18], which might eventually transform into a spatiotopic representation [19]. For example, there is evidence for presence of spatiotopic maps in MT [18,20] and lateral occipital cortex [21], but these studies are inconsistent with other research which supports retinotopy in all visual areas [16,22]. Retinotopic coding is even said to be the default or the ‘native’ coordinate system, in which most of the visual processing happens [23].

One interesting proposal for a neural explanation of visual stability is through remapping [24]. Retinotopic coordinates are known to be updated with each saccade based on the phenomenon of predictive remapping [25], where a neuron responds to a stimulus expected to fall in its postsaccadic receptive field, even before the saccade is executed. Remapping in neurons has been investigated in the parietal cortex [26], frontal eye fields (FEF) [27,28], superior colliculus (SC) [29] and the extrastriate visual cortex [25]. Research in these areas [26,30,31] suggests the presence of a saliency or priority map, driven by both bottom-up (stimulus driven) and top-down (behaviorally relevant) factors. Activation in this priority map may serve to guide the allocation of attention. The retinotopic image after a subsequent saccade can be predicted by updating attention pointers that are top-down connections from priority maps, such as lateral intraparietal cortex, FEF, and SC, to feature maps, such as MT, V1–V4, guiding spatial attention [32]. This hypothesis assumes the enhancement of visual processing at the attended location immediately before [8] the saccade and at its remapped location after the saccade [32].

Although a neural mechanism, remapping is still limited to attended targets [33], and this may still be sufficient to ensure a type of visual stability [24] since remapping can also occur across the whole visual field even for stimuli which are not saccade targets [34]. Recent studies [35,36] have confirmed an attentional mechanism supporting a link between neural and behavioral levels of remapping: horizontal transfer of activity in priority maps increases sensitivity at the remapped locations of attended targets in feature maps thus providing trans-saccadic tracking of attended targets. Attention may not only be necessary for remapping, but it may be attentional pointers that are remapped rather than the receptive fields themselves [37]. Importantly, a number of studies revealed that not only attentional pointers but also feature information is involved in trans-saccadic remapping [19,38]. Taken together, these results suggest that remapping and its behavioral correlates may show performance trade-offs as attention is manipulated across multiple tasks. For example, MacInnes and Hunt [11] used an attentional load manipulation and showed reduced accuracy on a post-saccade localization task when attention was loaded. What has not been shown is whether a similar tradeoff may be observed for remapping at the saccade landing location.

Additionally, if predictive activations of receptive fields support the stabilized perception of the visual scene, one might observe evidence of this prior to an eye movement, meaning that the perceived direction of gaze might shift to its future location prior to the eye movement that takes it there. This suggestion was supported in [9] where it was found that we seem to be looking at a new target before our eyes arrive there.

Another mechanism of maintaining information across saccades is trans-saccadic memory which allows to store the information about visual objects despite rapid changes in fixation points and update this information after each saccade [39,40,41]. Early evidence for the existence of trans-saccadic memory came from the work of Henderson et al. [39] who demonstrated an advantage to target identification if its preview was shown, suggesting that the target representation was retained in the memory across eye movements. Further properties of trans-saccadic memory suggest that both object identity and location could be maintained across a saccade for objects at [41] or near the saccade target [42].

In general, attention and VWM have both been suggested as mechanisms underlying trans-saccadic integration potentially due to the guidance of attentional pointers [37]: the results presented in Stewart, Shultz [43] suggest that if a location is considered relevant then it can receive both attention and memory resources to be kept during trans-saccadic integration. Although a great variety of studies refer these two processes as two distinct ones though highly overlapping, there is also an opposite view since they share the same capacity, the same control processes, the same content (see [44]), and may be different manifestations of the same underlying neural mechanisms [45]. For instance, holding a spatial location in working memory was shown to enhance the detection of stimuli presented at the memorized location [46].

### Proposal

In the current study, we use a dual task paradigm to examine the role of attention in remapping across saccadic eye movements. Although localization of peripheral targets across saccades involves both VWM and attention, only attention has been indicated in the predictive remapping of information at the future saccade location. Our study involved a dual task with the accuracy of both requiring spatial attention: reporting the hand position of a clock at the saccade target location (task 1) and reporting the perceived location of a peripheral stimulus (task 2). As a control condition, participants will maintain fixation while the clock is displaced to move to the location of the participant’s gaze.

By forcing participants to split resources between two tasks (localization task vs. clock task), we hypothesize a trade-off in performance for the two tasks. If attention is required for remapping in both saccadic condition tasks, then participants will be forced to prioritize one and this will result in reduced resources available for the other. Based on the results in [9], we expect the presence of a saccade during the clock task to result in the transfer of the clock face to the fovea or the shift of the apparent gaze direction to the clock face. Either (or both) of these will affect the time that participants see on the clock once they judge it to be under direct gaze. Specifically, they will see a time that is earlier than the actual time when gaze finally arrives at the clock. Importantly, this will happen to a lesser frequency or extent when attentional resources are focused on the second task and less available for the saccade and the remapping. We therefore further predict an interaction of saccade task by localization error with increasingly veridical clock estimates as the error in localization decreases. Since fixation trials do not require attention to execute the saccade, we expect the reports to be more accurate with a possible trend to reporting times after the clock has reached the endpoint of its travel, where it falls under direct gaze.

## 2. Materials and Methods

### 2.1. Participants

The initial sample included 23 healthy subjects (15 females, 22–26 years of age, mean age = 23; 8 males, 23–28 years of age, mean age = 24). Three subjects were excluded from analysis due to insufficient data (eye-tracker calibration problems, and/or not following the instructions). All participants reported normal or corrected-to-normal vision, without color blindness and provided informed written consent. A debriefing session was conducted, and participants’ questions were addressed; more details were provided in a written format, if requested by the participant.

### 2.2. Apparatus

Stimuli were presented on a 1920 × 1080 p screen in a dark room (refresh rate 144 Hz, screen width 54 cm). MATLAB 2011a with Psychtoolbox 3 package [47] was used to generate stimuli and record behavioral responses. A chinrest ensured a 76 cm viewing distance to the screen and minimized head movements. Eye movements were recorded with SR-Research EyeLink II system (SR Research, Mississauga, ON, Canada) at a temporal resolution of 1000 Hz. A five-point eye tracker calibration was performed twice in an experiment, before each block, and during blocks, if necessary.

### 2.3. Stimuli and Procedure

At the start of the experiment, instructions were provided to the participants, verbally and in written format. The experiment consisted of two blocks of 100 trials each, with the entire experiment lasting for about 45 min. On each trial, participants were instructed to make two, nontimed responses corresponding to the two tasks. The localization response was to report the remembered position of a small peripheral dot, and the clock response was to report the remembered position that a clock hand was on when it first arrived under the participant’s gaze.

At the start of each trial, the participants were presented with a black screen and a white fixation dot 3.5 degrees to the left of the center and an empty clock face of 1 degree width located 3.5 degrees to the right of the center. Participants performed a drift correction by pressing the space bar while fixating the left fixation dot, starting the trial as well as the clock spinning. Then, 100 ms after trial onset, a dot appeared in a location in the upper screen, randomly located within a nonvisible square of 5 degrees width, centered 7.5 degrees up from the screen center. The localization dot remained on the screen for 1000 ms while the gaze was restricted to fixation. A total of 400 ms after the dot’s removal, the second task began, and instructions depended on whether the task was in the saccade or fixation block of trials. In the saccade block, the fixation was removed, and the participant was tasked with making an immediate saccade to the center of the spinning clock. In the fixation condition, the clock was displaced toward the fixation dot and replaced the fixation point once it arrived. In the fixation condition, the clock was moved from its peripheral location toward fixation where it replaced the fixation dot once it arrived. In this case, the clock moved with a latency and duration randomly chosen from a distribution which matched saccadic latency and duration from a previous experiment (mean latency 225 ms, SD 95, duration 45 ms).

For both blocks, saccade and fixation, the participant was instructed to remember the location of the upper dot as well as the position on the clock hand when it was first seen directly under gaze. Reporting of both tasks occurred at the end of trial and participants were given as much time as needed to respond. The mouse cursor appeared for the response phase and participants were first asked to click the mouse at the remembered position of the dot in the upper hemifield. They were then presented with a new clock with the hand set to a random location and asked to use the up and down arrow keys to position the hand at the remembered location for that trial. 

**Figure 1:**
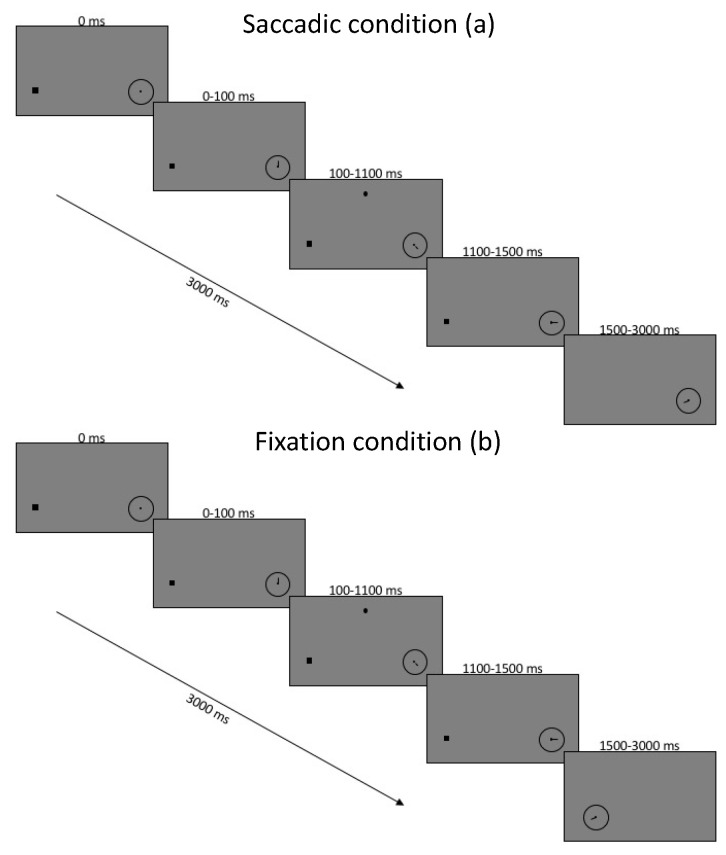
Timing of experiment conditions for saccade block of trials (**a**) and fixation block of trials (**b**). Images are not to scale.

Dependent variables were the error in Euclidean distance between target dot and mouse click response for the localization task and the error between reported and actual clock position for the clock task. The error for the target dot was the Euclidean distance between actual and reported dot location and reported in degrees visual angle (as used in [11]). The clock error was calculated as a signed error difference between the actual clock time at first direct gaze, and the time reported by the participant. All errors in the reported clock angle were analyzed in milliseconds with negative numbers representing a reporting error prior to veridical (the angle of the clock’s hand when it arrived at the fovea). Since the error on a circular clock face could be in either direction, the calculation assumed that the closer of the two signs was correct, since the larger would mean a remembered error of larger than 500 ms (more than half a complete rotation). The only independent variable was the blocked saccade manipulation, but subject was included as a random factor. The block sequence was counterbalanced between participants and the first 10 trials of each block were removed as practice trials. The participants had resting periods between blocks and as needed within each block. The time course of a sample trial is shown in Figure 1.

## 3. Results

Results were subjected to a linear mixed effect analysis using R and the LME4 package with subject as a random factor. The primary dependent variable was the error in clock response as measured in milliseconds. Fixed effects included Saccade condition (saccade or fixation) and error in Euclidean distance for the localization task. Fixed effects (intercepts and/or slopes) were tested for fit by a chi squared test using ANOVA in the CAR package. Reference level for the model used the saccade condition and veridical for error judgements.

**Figure 2:**
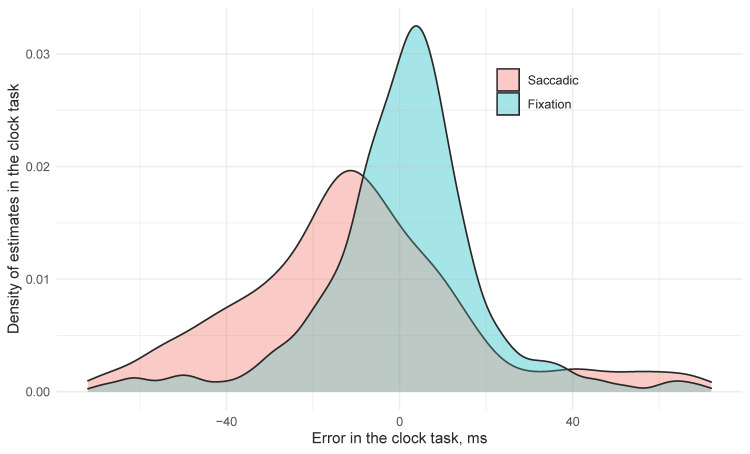
Distributions of saccadic and fixation clock error.

In the saccade condition, the mean error for clock position was −38.1 ms (SE 12.7) earlier than its orientation when it arrived at the fovea. In contrast, clock settings in the Fixation trials were on average 10.5 ms (SE 7.7) later than the correct time, and this difference (+48.6 ms, SE 10.0) was significant. The difference between two conditions is presented in Figure 2: in the fixation condition the answers in clock task were quite accurate with a slight tendency to delayed estimations. The saccadic condition was less accurate, but the participants tended to report clock angles that were earlier than the angle on the clock when their gaze landed on it. Euclidean error of probe responses also improved the model fit (χ2(1) = 9.3, *p* = 0.002) with larger errors at the probe resulting in earlier estimates of the clock (−10.6 ms per degree probe error, SE 2.6). Critically, we see a saccade by localization error interaction (χ2(1) = 7.4, *p* = 0.007) with saccadic trials resulting in 8.4 ms (SE = 3.1) of error for every additional dva of Euclidian localization error, suggesting that as attention to the localization task is reduced, predictive magnitude for the clock increased, but only in the saccade condition (t < 1.0 for the influence of Euclidean distance in the fixation condition).

**Figure 3:**
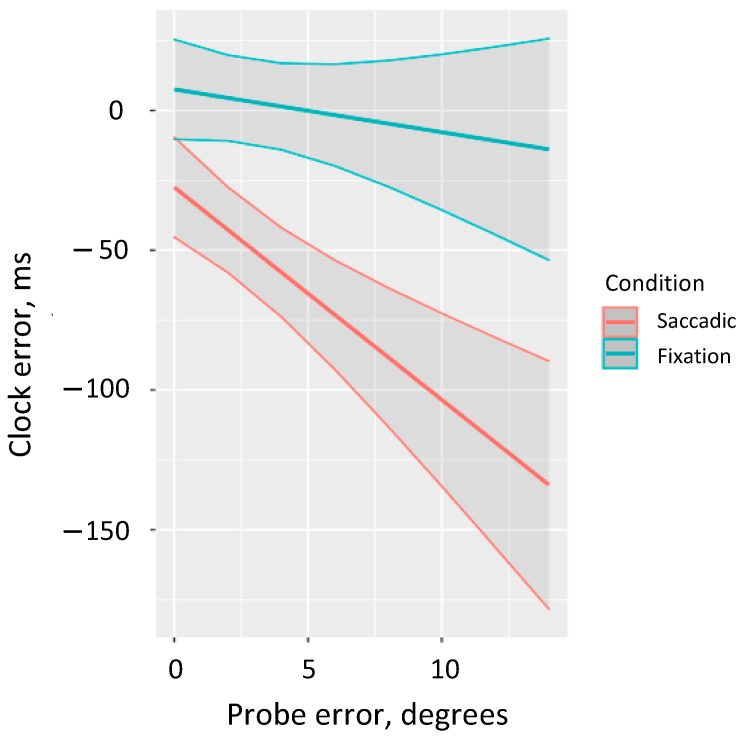
Predicted estimates of error in the clock task by the localization error for both saccadic and fixation conditions.

We performed a more detailed analysis of the interaction between saccade condition and Euclidean distance error to consider the marginal predicted performance in the clock task depending on the error size in the localization task (ggpredict from the package ggeffects [48]). The results revealed that accurate clock predictions are observed in the fixation condition across the full range of probe errors. By contrast, in the saccadic condition we observe decreased performance in the clock task and we observe greater negative clock errors as localization error increases (in the full range we observe underestimation) (Figure 3).

Finally, we tested the possibility that the accuracy of the saccadic landing position was a factor in clock estimation accuracy. For example, if increased attention to the localization task reduced the amount by which the clock estimate was in advance of the true landing time, then that increased attention will reduce saccade accuracy as well as reducing the remapping effect on reported time. We also tested the impact of the saccadic response time (SRT) and the saccadic duration to the fixation’s removal. Although our clock estimation error was based on the actual saccade landing time, the SRT and saccade duration may still influence predictive remapping. For example, task demands of the localization task may delay the onset of saccadic programming or execution after programming. Delays that impact the landing time of the saccade but not the time that the clock face was remapped could lead to a larger negative report. We did an additional lme analysis using data from the saccade condition with additional fixed effects of SRT, saccade duration and saccade landing position defined as the Euclidean distance between the center of the clock and the coordinates of the initial saccade toward the clock after the removal of fixation. We observed main effects of SRT (χ2(1) = 49.5, *p* < 0.001 and saccadic landing error (χ2(1) = 8.8, *p* = 0.003) with both slower SRTs and larger landing errors resulting in later clock estimates but no effect of saccadic duration (χ2(1) = 2.5, *p* = 0.11). Importantly, the error in probe localization was still a significant factor in clock estimation accuracy (χ2(1) = 11.0, *p* < 0.001), again with larger errors in localization resulting in earlier, predictive clock estimations. Additionally, none of saccade landing error (χ2(1) = 0.18, *p* = 0.180), saccadic duration (χ2(1) = 0.53, *p* = 0.462) nor the SRT (χ2(1) = 0.04, *p* = 0.832) showed a significant interaction with the localization error, suggesting that effects were independent.

## 4. Discussion

We used a dual task paradigm to test the role of attention in predictive remapping. A localization task measured the post saccadic reporting error of dot, while the clock task measured the reporting error of the perceived time that gaze landed on a spinning clock. We proposed that if remapping in the saccade condition required attention, then we would see an interaction between the two errors that was larger, or only in the saccade condition. When generating a saccade to the spinning clock, we observed a mean perceived arrival time that was earlier than the actual arrival time. In contrast, when the clock was displaced to the current gaze position, the estimated clock time was about equal to the actual. These findings are consistent with a previous study by Hunt and Cavanaugh [9] who observed a similar predictive error of 39 ms in the saccade condition suggesting that the subjective experience of fixating the clock hand precedes the moment of actual gaze arrival. One possible explanation for such anticipatory estimation is pre-saccadic remapping since this predictive error was only seen in the saccade condition. We also observed a significant interaction between the error of peripheral localization and task type on the magnitude of the clock prediction. Localization and predictive remapping likely share a resource since accuracy at localization reduces the magnitude of the predictive error, but only in the saccade condition. We did observe an effect of the saccade landing position on the clock estimation error, as well as an effect of the saccade latency after removing the fixation. These effects, however, did not interact with the impact of the dot estimation on the clock, so likely independent influences.

In Hunt and Cavanagh [9], participants also reported times in advance of the true time at saccade landing when making an eye movement to a spinning clock, and this effect remained robust across various speeds and directions of the clock hand. For example, when no hand information was available before the saccade, the reported time was seen to lag behind the actual in all conditions. Hunt and Cavanagh [9] proposed that the remapping of the saccade target back to the fovea just before the saccade creates the experience of looking straight at the clock while it has an earlier time than it will have when the eye actually lands on it. However, this shift of attention is unlikely to be the only reason for anticipation since it can move around the visual field covertly without eye movements. Instead, as proposed in Hunt, Cavanaugh [9] the more plausible interpretation is a preview of the saccade target caused by the remapping of the saccade target back to the fovea just prior to the saccade. This remapping transfers the activity from its current location to the one expecting after the eye movement that allows to maintain a representation of this new position across the saccade.

The results of the dot localization task can also be considered as consistent with MacInnes and Hunt [11] where participants performed a dual localization and vernier acuity task. By manipulating the attentional load, they revealed that the ability to keep track of locations across saccades depends, at least partially, on attention and varied according to the degree of the attention availability. They also demonstrated that the participants were able to flexibly use multiple frames of reference to compensate for those that are less reliable or less available. Remapping is likely only one of many sources of perisaccadic location information. It is also important that in their study both the localization and vernier acuity tasks had to be performed simultaneously prompting one to assume a dual task for attention. In the current work, however, the dot on the localization task appeared before the clock, meaning that short-term visual memory was required to encode the dot’s location, but we suggest that it was more likely attentional resources were required to update the coordinates in our saccade condition. In fact, the saccade did not seem to disrupt the localization task, at least as compared to our fixation condition (mean localization error saccade 2.9 degrees for saccades, 2.6 degrees for fixation clock). Additionally, it is possible that saccadic remapping is responsible for some degree of interference with spatial working memory [49], though the other work showed no dependency between visual attention and spatial memory performance when memorizing map routes [50]. In our results, however, we suggest that VWM was not a factor in the task, and our key interaction can be best explained with the competition of attentional pointers between the tasks’ locations/objects.

The results of the current study confirm that information from the saccade landing location is available prior to the saccade arrival time and this effect is represented by the subjective experience of looking at a target before the gaze actually lands on it. This predictive representation of the saccade target is likely a perceptual consequence of neural remapping and depends on allocation of available attention. Remapping provides a potential mechanism by which pre-saccadic and post-saccadic information may be maintained to provide a stable visual perception without the need for an explicit spatiotopic representation but requires sufficient attention to execute the predictive remapping.

## Data Availability

Data are available on request.
